# Has Pasteur Failed?

**Published:** 1890-12-27

**Authors:** 


### TURNING OVER NEW LEAVES.

### HAS PASTEUR FAILED?*

SINCE the days of Jenner no discovery in medical science has aroused more interest than M. Pasteur's theory of inoculation for hydrophobia and its apparent complete success. No experimenter could have chosen a subject more likely to captivate public interest; even consumption, regarding which we now await with interest the result of Dr. Koch's labours, has not such a hold on the public imagination. The myths of ages linger around hydrophobia, both the disease and its treatment. We are all familiar by repute with some of its victims—for example, the lovely and refined young girl who, bitten by a dog, forgets human speech and barks in canine fashion. No one we knew ever met her, or even one of her intimate friends or near relations, but her story goes from mouth to mouth, and grows as it proceeds. Even among doctors very few have seen a thoroughly unmistakable case of hydrophobia. With dog bites every practitioner has to deal, and with their sufficiently alarming results; but these results are not hydrophobia, but in most cases fear which might develop, into hysteria and madness. Tell some people that they are in danger of hydrophobia because a healthy, toothless puppy has made a snap at their hand, and immediately they will show all the traditional symptoms. "But they won't die of it," you may say. That is by no means certain. People die of fear, of shock, of grief, of many things which have not yet been traced to the ubiquitous microbe. On this point we may be permitted to narrate a true story, which, though it has nothing to do with hydrophobia, is not wholly irrelevant. A young doctor who had recently gone to India saw a native girl expectorating freely, and noticed that the saliva was of a deep red colour. Horrified at the sight, he went up to her, told her that she was suffering from hemorrhage from the lungs, that she was in great danger, and must go home at once. He went with her to warn her parents, and took all measures to relieve the alarming symptoms, but in spite of everything the girl died that evening. Subsequent post-mortem examination showed that there was nothing wrong with her lungs, and it finally transpired that when the doctor saw her she had been chewing betel-nut, to the juice of which was due the red stain that had so startled him. The girl had really died of fright, and the rash young doctor who had frightened her was directly responsible for her death.

Clinical teachers can tell how often medical students imagine themselves to be suffering from the diseases they hear about for the first time, imagination simulating all the symptoms; and we all know how periodic cancer scares arise among women, and in certain states of public opinion every trifling attack of eczema is raised to the grim dignity of leprosy. Rabies is a very rare disease, much rarer than cancer, or even leprosy, but it is much in the public mind, and, having regard to the nature of the public mind, we incline to hold with Dr. Dolan, whose little book on "Pasteur and Rabies" contains more fact and less fancy than most contributions to the subject, that it is "a by no means improbable thesis" that "cases may have been returned as hydrophobia, which really were delirium tremens, meningitis, mania, &c."

The statistics of hydrophobia have never been as carefully kept in England as in France, and as that is also the chief field of M. Pasteur's operations, it is in every way convenient that Dr. Dolan's figures should be largely drawn from that country. He quotes the statistics given by M. Dujardin-Beaumetz, a hygienist of undoubted position and probity, as to the deaths from hydrophobia for the Department of the Seine, including Paris, during the years 1880—89 inclusive. They are as follow: 1880, 4; 1881, 21; 1882, 9; 1883, 4; 1884, 3; 1885, 22; 1886, 3; 1887, 9; 1888, 19; 1889, 6; in all exactly a hundred, with regard to which several points are to be noted. First, that there seems to be a periodicity in hydrophobia; every four years or so the deaths increase amazingly during one season, and then fall to their former level. M. Pasteur's method has, as yet, had only a small effect on this. From 21 deaths in 1881 and 22 in 1885, we have only the small declension to 19 in 1888. The average mortality indeed seems to be little altered. For the years 1882-85 the total deaths amount to 38; for the years 1886-89 the deaths number 37. A note by M. Dujardin-Beaumetz on the year 1889 gives a special point to these statistics: "The observations of cases of rabies in Paris in 1889 include three children and three adults. Of the six sufferers who perished, three were under the anti-rabic treatment, and three were not taken to L' Institut Pasteur at all." Three were inoculated, three were not, and all six died! If Pasteur can show no better results than this, the utmost he can plead for his treatment is that it does no harm.

He may, however, say, and with reason, that sufferers from hydrophobia hasten to Paris now at a rate they never did before in order to receive his treatment. Such of these as die there swell the mortality of the department in lieu of that of their own parish, and thus confuse the statistics. Even so, this would be an admission of occasional failure, but the excuse is valid as far as it goes.

But other statistics go to prove that in France generally deaths from hydrophobia have not diminished since Pasteurism came in vogue. In 1863 Dr. Tardieu, in a report presented to the Minister of Hygiene, gave 25 cases per annum as the average mortality from hydrophobia in France. We have in Dr. Dolan's book statistics of the deaths from hydrophobia from 1850 to 1872 inclusive. They give an average mortality of rather less than 36. Taking Pasteur's own statistics for the year 1886, we find that the total deaths numbered 37—19 among the inoculated, 17 un-inoculated. In this table only French patients are counted, so it cannot be regarded as in any way misleading. Evidently, then, the mortality from hydrophobia in France is *in statu quo ante* Pasteur, a fact which, in a country with such a stationary population as France, affords much better ground for comparison than it would in England.

M. Pasteur, indeed, gives statistics very different from these—statistics which show a much heavier mortality from hydrophobia than appears from the figures we have quoted. Our figures are, however, official, and, having been in most cases collected in pre-Pasteurian days, they are not intended either to prove or to disprove any special hypothesis. In at least one case M. Pasteur's statistics are distinctly wrong. In a note communicated to the Academy of Medicine, in November, 1887, he says: "We know that 60 persons have died of rabies in the Paris hospitals during the last five years. A mean of 12 per year." The records of the hospitals do not verify this. They give a total of 26 deaths, or 5·2 per year.

Still, if M. Pasteur does just half as much good as his admirers claim, he will be no small benefactor to humanity; and this brings us to the question of the cures he has wrought. The word "cure" is, indeed, not wholly accurate. Inoculation is a prophylactic that is meant to prevent hydrophobia from developing rather than a remedy to operate when the disease has appeared. This makes computation a little difficult. If a man who has been bitten by a mad dog is inoculated and escapes hydrophobia, we cannot on that account say with any degree of certainty that inoculation has saved his life; for the question of his ever developing the disease would depend to a great extent on constitution,

*Pasteur and Rabies: By Thomas M. Dolan, M.D., F.R.C.S., Ed., London. (Messrs. George Bell and Sons, York Street, Covent Garden.)

temperament, and circumstances apart from the bite of the dog. Many persons bitten by dogs, which have not subsequently been proved to have been mad, have been treated by Pasteur. Has he the right to record these cases at all? One would say that it was at least necessary to prove that the animal was rabid when the injury was inflicted if the treatment is to be credited with working a cure. The bite of a healthy dog does not cause hydrophobia, and practitioners who have treated hundreds of dog-bites will admit that they have never seen a case of hydrophobia. If we could be sure that Pasteur's patients had all been bitten by rabid animals we should have something certain to go upon. The frequent lack of assured testimony on this point gravely affects the value of the statistics of cure.

Again, we have the failures, and the reasons given for them. That any human system should succeed in every case is too much to ask; but assuredly Pasteur does not wholly vindicate his cause. Most people still remember the death of Lord Doneraile, who was inoculated eleven days after being bitten by a pet fox, and subsequently died of hydrophobia. The reason assigned for his death is that too long an interval had elapsed between the injury and the treatment. If this explanation be accepted we must certainly take away from M. Pasteur and assign to the *vis medicatrix natura* all "cures" in which the treatment did not commence till after the eleventh day. This Pasteur does not do, but on the contrary, besides including such cases in his list of successes, he expressly declares his belief in the efficacy of inoculations beginning as late as a year after the patient has been bitten. Other mishaps are accounted for by the sufferers not having been treated on the "intensive" system, by which on every successive day of the treatment a spinal cord taken a day more recently from an inoculated rabbit is used for the injection. But Pasteur has himself modified this intensive process very largely on finding that patients treated by it were sometimes affected subsequently with paralytic rabies, the most fatal form of the disease.

In fact, it cannot be said that M. Pasteur has yet explained satisfactorily the causes of either his failures or his successes. It has been frequently pointed out in these columns that his method cannot claim the support of logical consistency. To set a weak virus to overcome a strong one that has already got a start of even a day is like bidding a child overtake a man. It can only be accomplished if the man is willing to wait for his pursuer, and we have no reason for assuming such an obliging disposition in the rabic virus. But we are far from saying that every process in medicine must be demonstrated by logic before we accept it. Let its practical value be proved to us, and it is enough. But we cannot hold that M. Pasteur has done this. That the consciousness of having been inoculated by him has saved many a poor soul from agonies of terror that might have resulted in hysteria or madness we freely and gladly admit, but more it would as yet be rash to say. M. Pasteur must revise his statistics. If he pleads delay in inoculation as an excuse for failure in one case, let him eliminate from his list of "cures" all in which successful results have been obtained after a period of equal length. If the use of the simple rather than the intensive method accounts for a death, the simple method can no longer be accredited with saving life. If M. Pasteur will publish these revised tables, and show at the same time a marked decrease in deaths from hydrophobia since his method was introduced, he may count on a fair hearing and an impartial verdict from doctors throughout the world. Till he does so he may win the voice of the public, but he can hardly expect the universal acclaim of the profession.

